# Kentucky State Dental Association

**Published:** 1861-05

**Authors:** A. L. Dwyer


### Proceedings of Societies.

### KENTUCKY STATE DENTAL ASSOCIATION.

TUESDAY, April 9, 1861.

THE Kentucky State Dental Association met pursuant to adjournment, at the Masonic Temple, at $10\frac{1}{2}$ o'clock, A. M.

The morning session was spent in general discussion, when the association adjourned until $2\frac{1}{2}$ o'clock, P. M.

The association met according to adjournment.

The proceedings of the morning session were read and approved.

The President called for reports of committees. The Executive and Examining Committees reported their duties performed, which reports were received and approved.

The Examining Committee reported the following gentlemen as applicants for membership, viz :

Drs. J. W. Baxter, Warsaw, Ky.; T. G. Lockerman, Louisville, Ky.; Geo. S. Jones, Russelville, Ky.; H. Baldwin, Elizabethtown, Ky.; H. S. Sanders, Lebanon, Ky.; A. L. Dwyer, B. A. Pierson, W. G. Redman, E. L. Green, Sam'l. Griffith, Louisville, Ky.; James L. Nourse, Cloverport, Ky.

A vote was then taken, and each applicant declared duly elected, after which they signed the constitution.

The Treasurer then made his report, which was received, and ordered to be placed on file.

TREASURER'S REPORT.

| | | |
|---|---|---|
| 1861.—April 9th—Amount in Treasury from last year | | $16 95 |
| Received as dues | | 40 00 |
| | | $56 95 |
| Expense incurred for printing | $ 6 75 | |
| " " for paper and pens | 90 | |
| " " for rent of hall in Masonic Temple... | 20 00 | $27 65 |
| Balance in Treasury | | $29 30 |

J. A. M'Clelland, *Treasurer.*

On motion of Dr. A. S. Talbert,

*Resolved*, That the medical profession of Louisville are hereby cordially invited to attend the sittings of this association.

The association then proceeded to the election of officers for the ensuing year. The President appointed Drs. Baldwin and Lockerman tellers. After balloting, the following officers were declared elected, viz :

W. D. Stone, *President.*

R. Peckover, *Vice-President.*

A. L. Dwyer, *Secretary.*

J. A. M'Clelland, *Treasurer.*

*Executive Committee.*—Sam'l. Griffith, H. Baldwin, T. G. Lockerman.

*Examining Committee.*—J. W. Baxter, W. G. Redman, Stoddard Driggs.

On motion of A. L. Dwyer, it was

*Resolved,* That we extend an invitation to all dentists, members of other societies, and to the dentists of this State, who may wish to become members of our association, to be present, and participate in the discussions.

On motion, a vote of thanks was returned Messrs. DuPont, for tickets and an invitation to visit the Artesian well.

Adjourned to meet at $7\frac{1}{2}$, P. M.

EVENING SESSION.

Association met pursuant to adjournment, and was called to order by the Chair.

Dr. Talbert then read an essay—subject, Professional success. Dr. Rogers also read a paper—subject, Filling decayed teeth. Dr. M'Clelland read an essay—subject, Extraction of teeth. At close of which, on motion of Dr. S. Griffith,

Adjourned till 9 o'clock, A. M. to-morrow.

MORNING SESSION.

Met at $9\frac{1}{2}$ o'clock, A. M. On motion, a vote of thanks was returned those gentlemen for essays read last night.

On motion, the essays were referred to the Executive Committee.

The Examining Committee then recommended the following gentlemen for membership, who were unanimously elected, viz: Drs. Edwd. Griffith, C. E. Dunn, G. B. Fitz and R. H. Wilson.

The President then announced that each gentleman would be limited to ten minutes, and not be allowed to speak the second time without the permission of the association. The President then announced the subject for discussion—Treatment of temporary teeth. This occupied the time to $12\frac{1}{2}$, P. M., when, on motion, adjourned to meet at 2 o'clock, P. M

AFTERNOON SESSION.

Met pursuant to adjournment. Discussion of treatment of temporary teeth continued. At 5, P. M., took up the next question in order, viz : Filling teeth.

Adjourned to meet at 7½, P. M.

EVENING SESSION.

Met at 8 o'clock, P. M. Discussion of the subject of filling teeth continued. After close of discussion, the meeting went into an irregular discussion on the causes of discoloration of teeth. On motion, adjourned to 9, A. M., to-morrow.

THURSDAY—MORNING SESSION.

Association met pursuant to adjournment, and opened with prayer by Dr. Sam'l. Griffith, when the meeting proceeded to the discussion of the subject of extracting teeth, at the close of which the President announced a recess of ten minutes, for the purpose of examining specimens of malformed teeth; also to examine instruments exhibited by Dr. J. T. Toland, of Cincinnati, O.

The meeting was then called to order, and proceeded to ballot for our next place of meeting. On counting votes, Louisville was declared the place selected. The President then tendered, on behalf of Silas Miller, Esq., proprietor of the Galt House, a room in that building for our use at our next meeting, which, on motion, was accepted.

The President then appointed the following gentlemen as delegates to the American Convention, to be held at Cleveland, Ohio, the last Tuesday in July next, viz : Drs. W. G. Redman, B. A. Pierson, Stoddard Driggs, and H. Baldwin. On motion, Dr. Talbert took the chair, and appointed Dr. W. D. Stone as one of the delegates.

The meeting then proceeded to discuss the subject of tobacco—its effects upon the health of the mouth. After the discussion, on motion, adjourned to meet at 2'clock, P. M.

EVENING SESSION.

Met pursuant to adjournment, at $2\frac{1}{2}$ o'clock, P. M., and proceeded to discuss the subject of "Artificial Dentures," after which, on motion, the President appointed Drs. M'Clelland, Pierson and E. Griffith as a committee to draw up and present to this association a resolution or by-law, for the better protection of this society against objectionable persons, which committee reported as follows;

*Resolved*, That dentists, not members of this association, to be admitted to its discussions, must be introduced by one of its members, and that no one who has been in the study and practice of dentistry less than two years be admitted, except such person be a student of one of the members of this society. Adopted.

On motion, the society went into discussion on Miscellaneous Subjects.

The President appointed as essayists for our next meeting Drs. Nourse, Baxter and Lockerman.

A vote of thanks was returned B. M. Patten, Esq., Director of the Kentucky Institute for the education of the Blind, for his kind invitation to visit that Institution. A vote of thanks was also given to Dr. J. T. Toland for his kindness in acting as reporter of this association. Also, a vote of thanks to our worthy President, Dr. W. D. Stone, for the able manner in which he had conducted the sittings. On motion, a vote of thanks was returned Dr. Goldsmith for the tender of the hall of the Kentucky School of Medicine for our use.

The Minutes were read and approved.

Prayer was then made by Dr. Sam'l. Griffith, and, on motion, the association adjourned to meet in this city the second Tuesday in April, 1862, at the Galt House.

A. L. DWYER, *Sec'y.*

